# Incremental predictive value of multimodal over unimodal assessment for upper limb motor recovery after stroke: a systematic review and meta-analysis

**DOI:** 10.3389/fneur.2026.1845257

**Published:** 2026-10-28

**Authors:** Ye Chang-Qing, Wang Yun-Lei, Chen Yu-Dong, Ma Lin, Du Xiao-Xia, Wu Xiao-Li

**Affiliations:** 1 Department of Neurology Rehabilitation, Beijing Bo'ai Hospital, China Rehabilitation Research Center, Beijing, China; 2 Capital Medical University School of Rehabilitation Medicine, Beijing, China

**Keywords:** biomarkers, meta-analysis, motor recovery, multimodal assessment, prognosis, stroke, upper extremity

## Abstract

**Background:**

Clinical scales predict post-stroke motor recovery but are insensitive to neural substrates. Neuroimaging and neurophysiological biomarkers capture corticospinal integrity, yet their incremental value over clinical assessment remains unquantified.

**Objective:**

To quantify the pooled incremental predictive value (ΔAUC) of adding biomarkers to clinical models for upper limb motor recovery at 3–6 months post-stroke.

**Methods:**

We systematically searched PubMed, Embase, and Web of Science from inception to January 31, 2026, for prospective cohort studies evaluating ≥2 assessment modalities. Random-effects meta-analysis pooled ΔAUC between multimodal and clinical-only models. Sensitivity analyses used alternative between-study variance estimators and the Hartung-Knapp adjustment. Meta-regression examined sources of heterogeneity. Risk of bias was assessed using QUIPS.

**Results:**

Twenty-three studies (*N* = 1,341) were included. Multimodal assessment significantly improved prognostic accuracy over clinical models alone (pooled ΔAUC = +0.112, 95% CI 0.061 to 0.163, *p* < 0.001). MEP status showed the largest incremental gain (ΔAUC = +0.150, 95% CI 0.023 to 0.277; *I*^2^ = 71.0%). Ipsilesional CST FA was reported in one study (ΔAUC = +0.220, 95% CI 0.110 to 0.330). Other DTI-derived metrics did not reach statistical significance (ΔAUC = +0.074, 95% CI −0.006 to 0.155). Task-based fMRI showed promise in one study (ΔAUC = +0.190, 95% CI 0.088 to 0.292). EEG, kinematic parameters, and resting-state fMRI could not be pooled. Stroke phase was a significant moderator (*β* = −0.127, 95% CI −0.247 to −0.007, *p* = 0.039), with larger gains in subacute versus acute assessments.

**Conclusion:**

In this systematic review and meta-analysis, adding selected biomarkers to clinical assessment was associated with improved discrimination for upper limb motor recovery after stroke. MEP status showed the largest incremental gain. Stroke phase significantly moderated the incremental value, with larger gains in the subacute phase. However, given the small number of studies and the prediction interval crossing zero, these findings should be considered statistically promising but not yet robust enough to guide routine clinical practice. Further validation in larger prospective cohorts is needed before MEP-guided stratification can be applied clinically.

**Systematic review registration:**

https://www.crd.york.ac.uk/PROSPERO/view/CRD420261288506, Identifier: CRD420261288506.

## 1 Introduction

### 1.1 The prognostic gap in post-stroke upper limb recovery

Accurate prediction of upper limb motor recovery after stroke is essential for personalized rehabilitation and patient counseling. The Fugl-Meyer Assessment for Upper Extremity (FMA-UE) is the most widely validated clinical tool for assessing motor impairment, alongside activity-based measures such as the Action Research Arm Test (ARAT). Yet baseline FMA-UE scores explain only 40–50% of outcome variance at 3–6 months (1–3). It’s prognostic ceiling is well documented: floor effects in severely impaired patients and ceiling effects in mild cases limit sensitivity (4, 5), and the scale provides no direct information about neural substrates governing recovery potential (6).

### 1.2 Biomarkers of corticospinal integrity and cortical reorganization

Advances in neuroimaging and neurophysiology have identified candidate biomarkers that capture these missing neural dimensions. Diffusion tensor imaging (DTI)-derived fractional anisotropy (FA) of the ipsilesional corticospinal tract (CST) quantifies white matter integrity and predicts motor outcomes (7–9). Transcranial magnetic stimulation (TMS)-elicited motor evoked potentials (MEPs) provide a functional assay of CST excitability; MEP presence is among the strongest predictors of favorable recovery (10–12). The PREP algorithm pioneered integrating MEP status and DTI-FA into a clinically actionable framework (13). Electroencephalography (EEG) oscillatory power and functional MRI (fMRI) connectivity patterns further characterize cortical reorganization (14, 15). Recent work using functional near-infrared spectroscopy has revealed that cortical activation during fine motor tasks correlates with bimanual coordination in high-functioning stroke survivors,underscoring the potential of task-specific neurophysiological measures to capture neural responses relevant to recovery (16, 17).

### 1.3 The rise of multimodal predictive models: promise and persistent uncertainty

Building on evidence that individual biomarkers correlate with motor outcomes, a growing number of studies have integrated two or more modalities into multivariable predictive models (9, 13, 18). These models combine clinical baselines (e.g., FMA-UE, age) with biomarkers from structural MRI, including ipsilesional CST fractional anisotropy and other DTI-derived metrics such as local diffusion homogeneity, lesion load, or whole-brain volumes, fMRI, TMS-MEP, EEG, or wearable sensors (9, 13, 18, 19). Across single-center cohorts, such models report excellent discriminative performance, with AUC frequently exceeding 0.85 (9, 13, 20), and machine learning approaches have further enhanced predictive accuracy (21–23).

Understanding the mechanistic underpinnings of these biomarkers—particularly their relationship to axonal regeneration, cortical remapping, and activity-dependent plasticity—is critical for interpreting their prognostic value and guiding the development of targeted neuromodulatory interventions (24, 25). Despite this prolific publication record, three fundamental uncertainties prevent clinical translation. First, most studies report only final model performance without testing whether adding biomarkers yields statistically or clinically significant improvement over a simpler clinical baseline (26). Second, extreme heterogeneity across studies has precluded meta-analytic synthesis of the average incremental gain from specific biomarkers (27). Third, it remains unknown whether more modalities are always better; once CST integrity and excitability are characterized, additional measures like resting-state fMRI may provide diminishing returns (9, 13). Conversely, a parsimonious “core set” of two or three biomarkers may achieve near-maximal accuracy (24). These three gaps form the central rationale for the present systematic review and meta-analysis (24, 26).

### 1.4 Study aims

To address these gaps, we conducted a systematic review and meta-analysis with the following objectives:

1. To quantify the pooled incremental predictive value (ΔAUC) of multimodal versus unimodal clinical assessment for upper limb motor recovery at 3–6 months post-stroke.
2. To identify which biomarkers provide the largest and most consistent incremental gains.
3. To explore sources of heterogeneity that moderate incremental value.

## 2 Methods

### 2.1 Protocol and registration

This systematic review and meta-analysis was conducted in accordance with the PRISMA 2020 statement. The protocol was prospectively registered with PROSPERO (CRD420261288506) on 13 February 2026, before the start of the literature search and data extraction. During the conduct of the review, we made minor updates to the eligibility criteria, risk-of-bias tool, and data synthesis methods to reflect the final analysis. These updates are documented in the PROSPERO record (Version 2.0). The registration and the final manuscript are now fully consistent.

### 2.2 Search strategy

We systematically searched PubMed, Embase, and Web of Science from inception to January 31, 2026. The search strategy combined terms covering: (1) stroke, (2) upper limb motor function, and (3) predictive models, with an emphasis on multimodal assessment. Methodological filters for predictive studies were applied, supplemented with machine learning terms. The full search strings for all databases are detailed in Supplementary Material 1. We also manually screened reference lists of included studies and relevant reviews. The search was restricted to English-language articles.

### 2.3 Eligibility criteria

Studies were included if they met the following PICOS (Participants, Interventions, Comparators, Outcomes, Study Design) criteria:

*Population*: Adults (≥18 years) with ischemic or hemorrhagic stroke at any phase (acute: <7 days; subacute: 7–90 days; chronic: >90 days).

*Index model*: Multivariable models incorporating ≥2 assessment modalities from different domains (clinical, neuroimaging, neurophysiological, kinematic).

*Comparator*: A unimodal reference model using only clinical scales or demographic variables.

*Outcome*: Upper limb motor function measured by a standardized scale (FMA-UE, ARAT, etc.) at ≥1 month post-stroke; primary outcome was FMA-UE at 3–6 months.

*Study design*: Prospective cohort studies. RCTs were considered eligible only if baseline predictive modeling data were reported separately; however, no eligible RCTs were identified.

*Exclusion criteria*: Cross-sectional studies; univariable associations only; insufficient data to calculate effect sizes; conference abstracts, case reports, reviews, editorials, or animal studies.

### 2.4 Study selection and data extraction

Two reviewers (YC-Q, WY-L) independently screened titles and abstracts using Covidence, followed by full-text review. Disagreements were resolved through consensus or consultation with a third reviewer (CY-D).

Data extraction was performed independently in duplicate using a standardized form. Extracted items included: study characteristics, predictors, outcome measures, modeling approach, and performance metrics (AUC, *R*^2^). When multiple models were reported, we extracted data for (1)the clinical-only baseline model, (2) intermediate models adding individual biomarker classes (where available), and(3)the full multimodal model. Complete extracted data are provided in Supplementary Material 2.

### 2.5 Risk of bias assessment

Two reviewers independently assessed methodological quality using the QUIPS (Quality in Prognosis Studies) tool. Before formal assessment, both reviewers calibrated their judgments on three randomly selected studies. Domain-level ratings (low, moderate, high) were assessed according to QUIPS guidance and are presented graphically using the robvis R package. Complete domain-level ratings for each study are provided in Supplementary Material 3. For sensitivity analyses, we pre-specified restricting quantitative synthesis to studies rated low or moderate risk in ≥4 domains and not rated high in “prognostic factor measurement” or “outcome measurement.” The detailed QUIPS ratings identified three studies with high risk of attrition bias, consistent with the domain summary presented in Supplementary Material 3 and Supplementary Figure 1.

### 2.6 Outcome measures and effect size extraction

The primary outcome was incremental predictive value, operationalized as the difference in area under the curve (ΔAUC) between multimodal and unimodal clinical models. When studies directly reported ΔAUC, these were extracted directly. When only individual model AUCs were reported, ΔAUC was calculated as the difference. For studies not reporting AUC, we converted odds ratios to Cohen’s d and subsequently to AUC using established formulas (28), or extracted correlation coefficients from R^2^, t-, or F-statistics using standard methods (29).

For studies reporting multiple follow-up time points, we prioritized assessments closest to 6 months (range 3–6 months). All effect sizes were transformed to a common metric (AUC or Fisher’s z). When 95% confidence intervals (CIs) were not reported, they were estimated using standard errors derived from sample sizes, with standard formulas applied for ΔAUC. Detailed conversion formulas and complete extracted data are provided in Supplementary Material 2 (Data extraction and effect size conversion).

### 2.7 Data synthesis and meta-analysis

We performed random-effects meta-analyses using the DerSimonian–Laird method (30). Heterogeneity was quantified using the *I*^2^ statistic (31). Sensitivity analyses were conducted using alternative between-study variance estimators (REML and Paule-Mandel) and the Hartung-Knapp adjustment for small-sample confidence intervals; prediction intervals were reported where defensible.

For the primary analysis, we pooled ΔAUC values from 7 studies reporting both clinical-only and multimodal model performance. For secondary analyses, we stratified studies by biomarker type (MEP, ipsilesional CST FA, other DTI-derived metrics, fMRI, EEG, kinematic). When studies contributed multiple biomarker comparisons, each comparison was treated separately, with sensitivity analyses to assess potential unit-of-analysis bias. For the secondary outcome, a separate meta-analysis of ΔR^2^ was performed across 7 studies.

Meta-regression was conducted to examine prespecified moderators: stroke phase (acute vs. subacute), outcome timing (3 vs. 6 months), and analytic method (machine learning vs. conventional regression). All analyses were performed in R (version 4.3.1) using the metafor package. Statistical significance was set at *p* < 0.05 (two-tailed).

### 2.8 Subgroup and sensitivity analyses

Pre-specified subgroup analyses were conducted according to: (1) biomarker modality type; (2) stroke phase; (3) outcome measure; and (4) risk of bias. For the DTI subgroup, studies reporting ipsilesional CST fractional anisotropy (CST FA) were analysed separately from those reporting other DTI-derived metrics (e.g., local diffusion homogeneity, lesion load, whole-brain volumes).

Sensitivity analyses tested robustness by: (1) excluding studies with high risk of bias in ≥2 QUIPS domains; (2) restricting to studies reporting AUC directly; (3) using a fixed-effects model; (4) leave-one-out analysis; and (5) using alternative between-study variance estimators (REML and Paule-Mandel) and the Hartung-Knapp adjustment for small-sample confidence intervals, with prediction intervals reported where defensible.

### 2.9 Publication Bias

Funnel plot asymmetry was assessed visually and using Egger’s regression test for the primary ΔAUC meta-analysis (32). A *p*-value <0.10 was considered indicative of significant asymmetry. When asymmetry was indicated, the trim-and-fill method was applied (33). For the primary ΔAUC analysis, Egger’s test showed no significant asymmetry (*p* = 0.278) and trim-and-fill did not impute any missing studies. For the secondary ΔR^2^ analysis, trim-and-fill imputed three hypothetical studies. Contour-enhanced funnel plots were generated to aid interpretation. Egger’s test was not performed for the secondary ΔR^2^ analysis because the number of studies was less than 10, as recommended by Sterne et al. (34).

## 3 Results

### 3.1 Study selection and characteristics

The systematic search yielded 1,847 unique records after duplicate removal. Following screening, 135 full-text articles were assessed, and 23 studies comprising 1,341 stroke patients met the inclusion criteria (Figure 1). All 23 included studies were prospective cohort studies. Supplementary Table 1. summarizes study characteristics. Sample sizes ranged from 19 to 207 (median 47, IQR 35–67). Eleven studies enrolled patients in the acute phase (<7 days), 8 in the subacute phase (7–90 days), 3 in the chronic phase (>90 days), and 1 included patients across acute, subacute, and chronic phases. The primary outcome was FMA-UE at 3 or 6 months in 17 studies; 3 studies used ARAT, 1 study used MFT, and the remaining 2 studies used other classification systems (e.g., 3-group classification, motor outcome). All 23 studies reported performance metrics for both clinical-only and full multimodal models. Nine studies (39%) further reported intermediate models incorporating individual biomarker classes.

**Figure 1:**
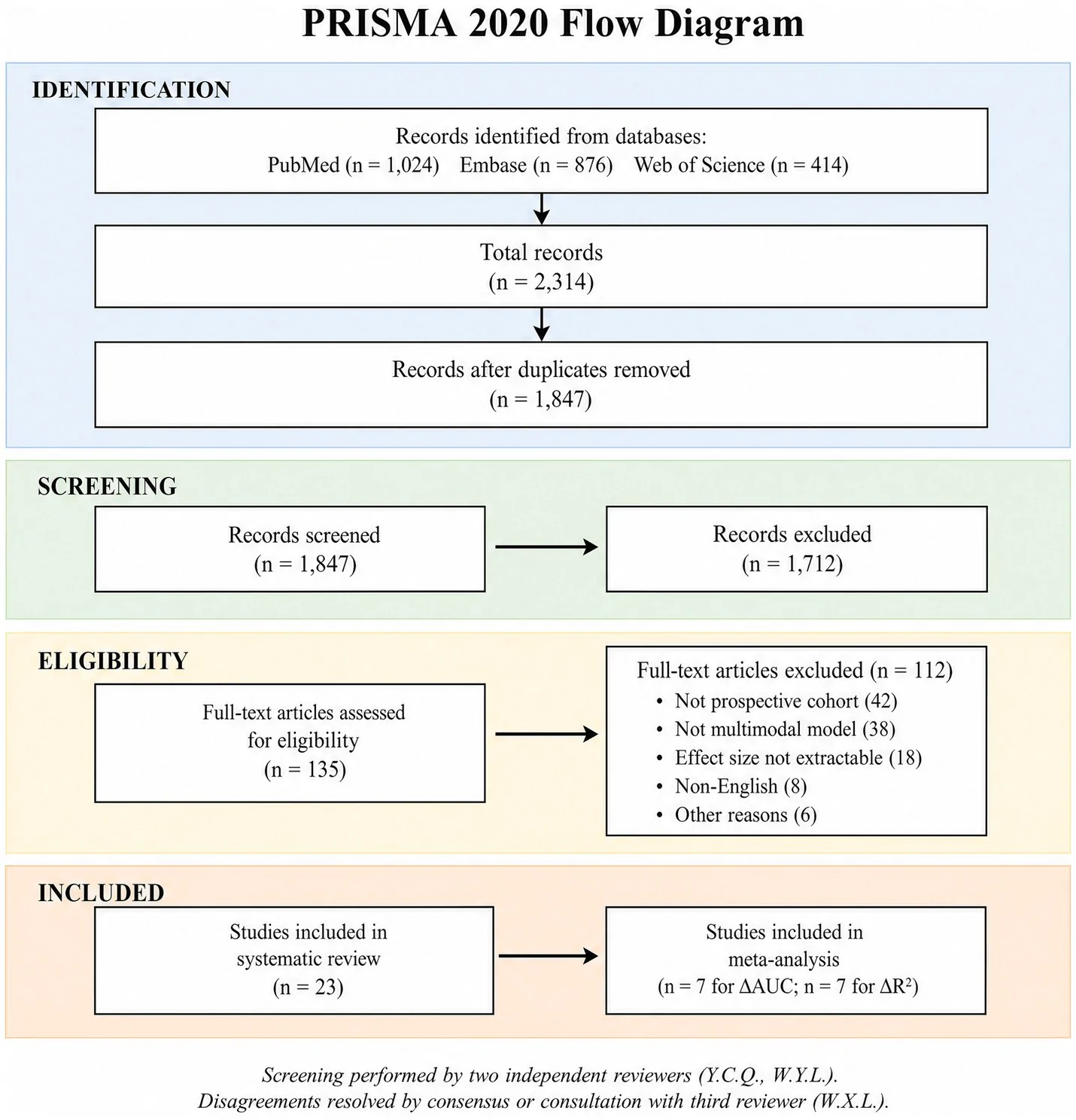
PRISMA 2020 flow diagram of study selection. A total of 2,314 records were identified through database searching (PubMed: *n* = 1,024; Embase: *n* = 876; Web of Science: *n* = 414). After removal of 467 duplicates, 1,847 unique records were screened. Following title and abstract screening, 1,712 records were excluded, leaving 135 full-text articles assessed for eligibility. Of these, 112 articles were excluded (not prospective cohort: *n* = 42; not multimodal model: *n* = 38; effect size not extractable: *n* = 18; non-English: *n* = 8; other reasons: *n* = 6). A total of 23 prospective cohort studies comprising 1,341 stroke patients met the inclusion criteria and were included in the systematic review. Of these, 7 studies contributed to the primary ΔAUC meta-analysis and 7 studies contributed to the secondary ΔR^2^ meta-analysis. Note: The number 1,847 refers to unique records after deduplication, not the number of patients. The total number of patients across the 23 included studies is 1,341. Screening was performed by two independent reviewers (YC-Q, WY-L). Disagreements were resolved by consensus or consultation with a third reviewer (WX-L).

### 3.2 Risk of bias

QUIPS assessment indicated low or moderate risk of bias in 20 studies. Domain 2 (Study Attrition) contained three studies with high risk of attrition bias. The most common source of potential bias was study attrition, followed by outcome assessor blinding and adjustment for confounders. In sensitivity analyses, excluding studies with high risk of bias in ≥2 QUIPS domains did not materially change the pooled ΔAUC. Summary risk of bias is presented in Supplementary Figure 1, with detailed domain-level ratings in Supplementary Figure 2.

### 3.3 Incremental predictive value: multimodal vs. unimodal models

Seven studies (*n* = 295) reported ΔAUC for both clinical-only and multimodal models. The pooled ΔAUC was +0.112 (95% CI 0.061 to 0.163; *p* < 0.001), with moderate heterogeneity (*I*^2^ = 52.4%, τ^2^ = 0.0024; Figure 2). The prediction interval was −0.026 to 0.250.

**Figure 2:**
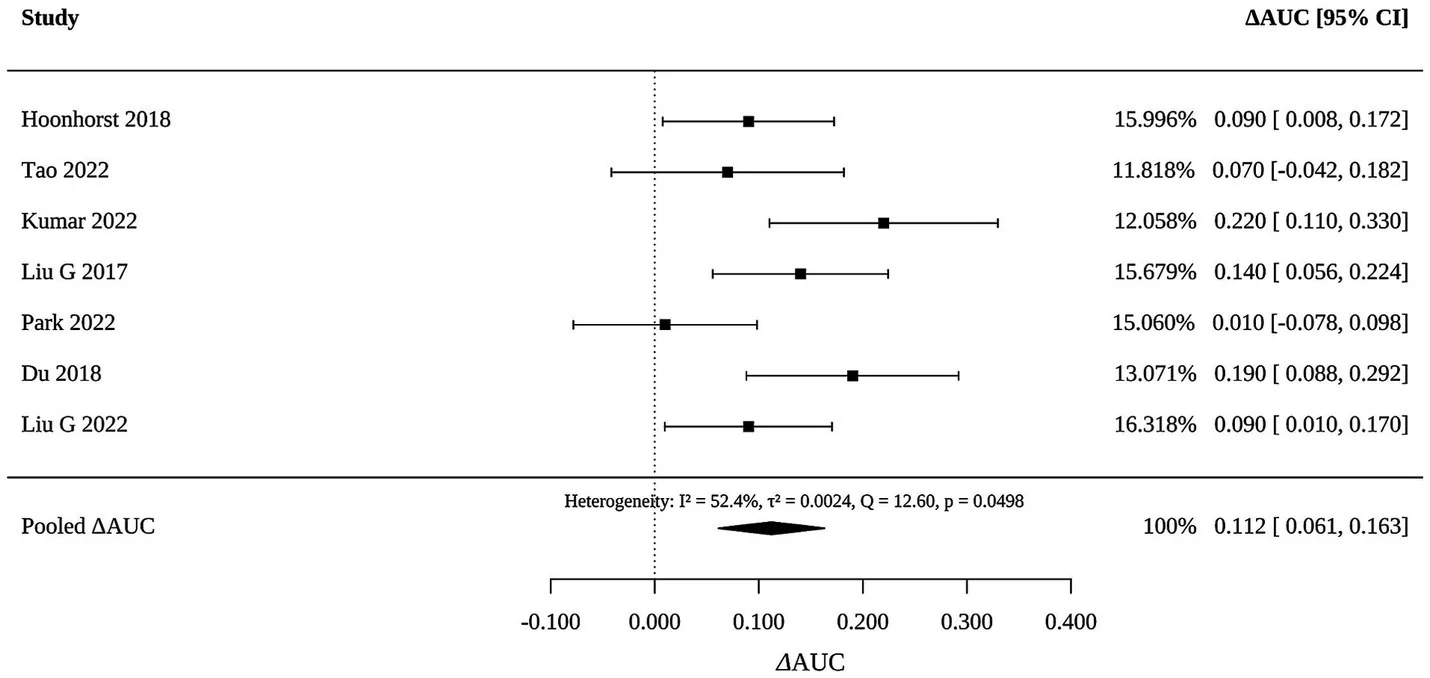
Forest plot of the incremental predictive value (ΔAUC) for multimodal versus clinical-only models. Seven studies (*n* = 295) contributed to the primary analysis. Squares represent study-specific ΔAUC estimates with 95% confidence intervals; the diamond indicates the pooled random-effects estimate (ΔAUC = 0.112, 95% CI 0.061 to 0.163; *p* < 0.001). Moderate heterogeneity was observed (*I*^2^ = 52.4%, τ^2^ = 0.0024, *Q* = 12.60, *p* = 0.0498). Weights are shown as percentages. The vertical dashed line indicates no incremental predictive value (ΔAUC = 0).

For seven studies (*n* = 355) reporting *R*^2^ values, the pooled ΔR^2^ was 0.175 (95% CI 0.082–0.269; *p* = 0.0002), with substantial heterogeneity (*I*^2^ = 91.8%, τ^2^ = 0.0136, Q = 72.89, *p* < 0.0001; Figure 3). The prediction interval was −0.072 to 0.422.

**Figure 3:**
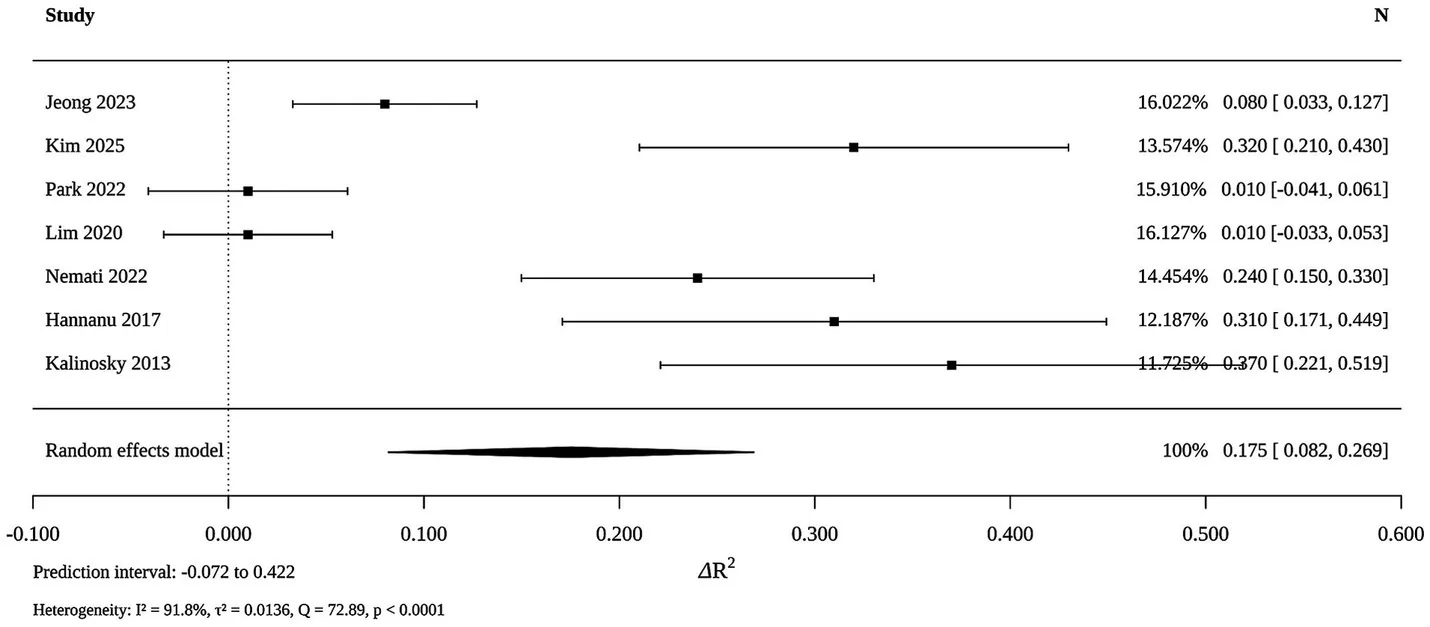
Forest plot of the incremental *R*^2^ (ΔR^2^) for multimodal versus clinical-only models. Seven studies (n = 355) contributed to the secondary analysis. Squares represent study-specific ΔR^2^ estimates with 95% confidence intervals; the diamond indicates the pooled random-effects estimate (ΔR^2^ = 0.175, 95% CI 0.082 to 0.269; *p* = 0.0002). Substantial heterogeneity was observed (*I*^2^ = 91.8%, τ^2^ = 0.0136, Q = 72.89, *p* < 0.0001). The prediction interval was −0.072 to 0.422. Weights are shown as percentages. The vertical dashed line indicates no incremental predictive value (ΔR^2^ = 0).

### 3.4 Biomarker-specific incremental value

Stratified meta-analyses by modality type were performed for studies reporting ΔAUC values (Table 1; Figure 4). MEP status provided the largest gain (ΔAUC = +0.150, 95% CI 0.023 to 0.277, *I*^2^ = 71.0%), based on 2 studies. Among the subgroups that could be pooled, only MEP status showed a statistically significant pooled ΔAUC. Ipsilesional CST fractional anisotropy was reported in only one study, with a ΔAUC of +0.220 (95% CI 0.110 to 0.330); because only one study reported this metric, pooled meta-analysis was not appropriate. Other DTI-derived metrics (e.g., FAAI or rFA, LDH, and lesion load) were reported in three studies, with a pooled ΔAUC of +0.074 (95% CI −0.006 to 0.155; *I*^2^ = 54.3%), which did not reach statistical significance. Task-based fMRI activation, evaluated in a single study, demonstrated a ΔAUC of +0.190 (95% CI 0.088 to 0.292).

**Table 1:** Biomarker -Specific Incremental Predictive Value (ΔAUC).

Biomarker added to clinical model	k (studies)	Pooled ΔAUC (95% CI)	*I* ^2^
MEP status (present/absent)	2	+0.150 (0.023 to 0.277)	71%
ipsilesional CST FA	1	+0.220 (0.110 to 0.330)	NA
Other DTI-derived metrics (FAAI/rFA, LDH, lesion load)	3	+0.074 (−0.006 to 0.155)	54.3%
Task-based fMRI activation	1	+0.190 (0.088 to 0.292)	NA
EEG power/coherence	Insufficient data	Not estimable	NA
Resting-state fMRI connectivity	Insufficient data	Not estimable	NA
Kinematic (wearable sensors)	Insufficient data	Not estimable	NA

ΔAUC, incremental area under the curve; MEP, motor evoked potential; CST FA, corticospinal tract fractional anisotropy; DTI, diffusion tensor imaging; FAAI/rFA, fractional anisotropy asymmetry index/relative fractional anisotropy; LDH, local diffusion homogeneity; fMRI, functional magnetic resonance imaging; EEG, electroencephalography; NA, not applicable. Data are pooled ΔAUC (95% CI) unless otherwise indicated.

**Figure 4:**
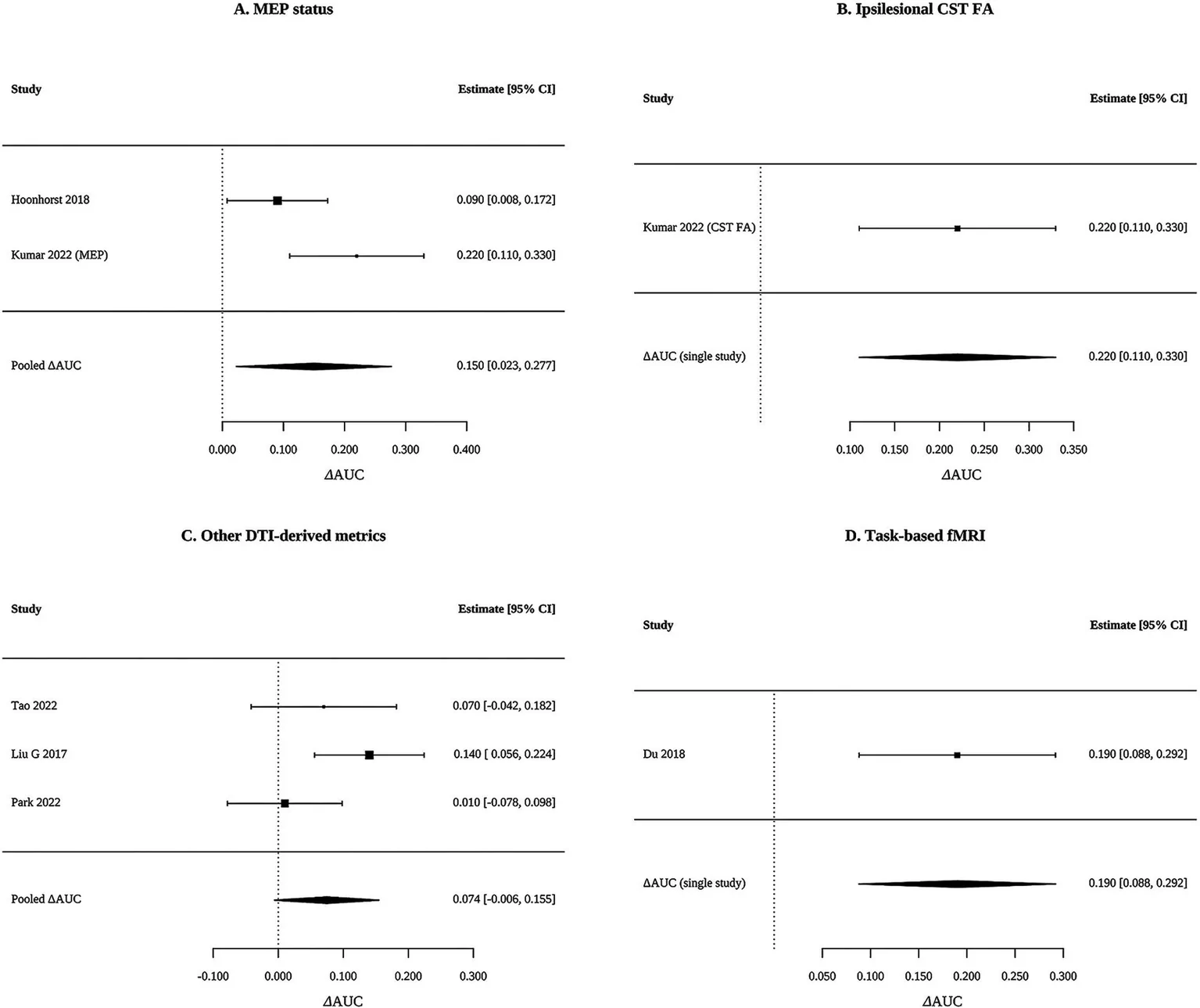
Biomarker-specific incremental predictive value (ΔAUC) for multimodal versus clinical-only models. Studies were stratified by biomarker type: **(A)** MEP status (2 studies; pooled ΔAUC = +0.150, 95% CI 0.023 to 0.277; *I*^2^ = 71.0%); **(B)** ipsilesional CST fractional anisotropy (1 study; ΔAUC = +0.220, 95% CI 0.110 to 0.330); **(C)** other DTI-derived metrics (3 studies; pooled ΔAUC = +0.074, 95% CI − 0.006 to 0.155; *I*^2^ = 54.3%; not statistically significant); **(D)** task-based fMRI activation (1 study; ΔAUC = +0.190, 95% CI 0.088 to 0.292). Squares represent study-specific ΔAUC estimates with 95% confidence intervals; diamonds indicate pooled estimates. The vertical dashed line indicates no incremental predictive value (ΔAUC = 0).

For EEG, kinematic parameters, and resting-state fMRI, we were unable to perform pooled meta-analyses due to insufficient reporting of ΔAUC. Individual studies reported promising results using alternative metrics—for example, Kim et al. reported a ΔR^2^ of 0.32 for EEG, and Zhou et al. reported a final multimodal model AUC of 0.86 for fNIRS-based kinematic assessment—but the absence of directly comparable ΔAUC values precluded quantitative synthesis.

### 3.5 Sources of heterogeneity

Meta-regression was conducted to examine prespecified moderators of incremental predictive value. Stroke phase was a statistically significant moderator (*β* = −0.127, 95% CI −0.247 to −0.007, *p* = 0.039), with larger ΔAUC for subacute-phase (7–90 days) compared with acute-phase (<7 days) measurements (Table 2, Figure 5). Outcome timing (*β* = −0.006, 95% CI −0.046 to 0.033, *p* = 0.756) and analytic method (*β* = −0.072, 95% CI −0.209 to 0.064, *p* = 0.301) were not significant moderators. The model explained 44.76% of between-study heterogeneity (QM = 5.08, df = 3, *p* = 0.166).

**Table 2:** Meta-regression analysis of prespecified moderators for incremental predictive value (ΔAUC).

Moderator	Coefficient	SE	Lower CI	Upper CI	*p*-value
Stroke Phase (subacute vs. acute)	−0.127	0.061	−0.247	−0.007	0.039
Outcome timing (per 1 month)	−0.006	0.020	−0.046	0.033	0.756
Analytic Method (ML vs. conventional)	−0.072	0.070	−0.209	0.064	0.301
Intercept	0.181	0.095	−0.005	0.367	0.057

*R*^2^ = 44.76%, QM = 5.08 (df = 3), *p* = 0.166, τ^2^ = 0.0014, *I*^2^ = 35.87%. ML, machine learning; vs., versus; CI, confidence interval; SE, standard error.

**Figure 5:**
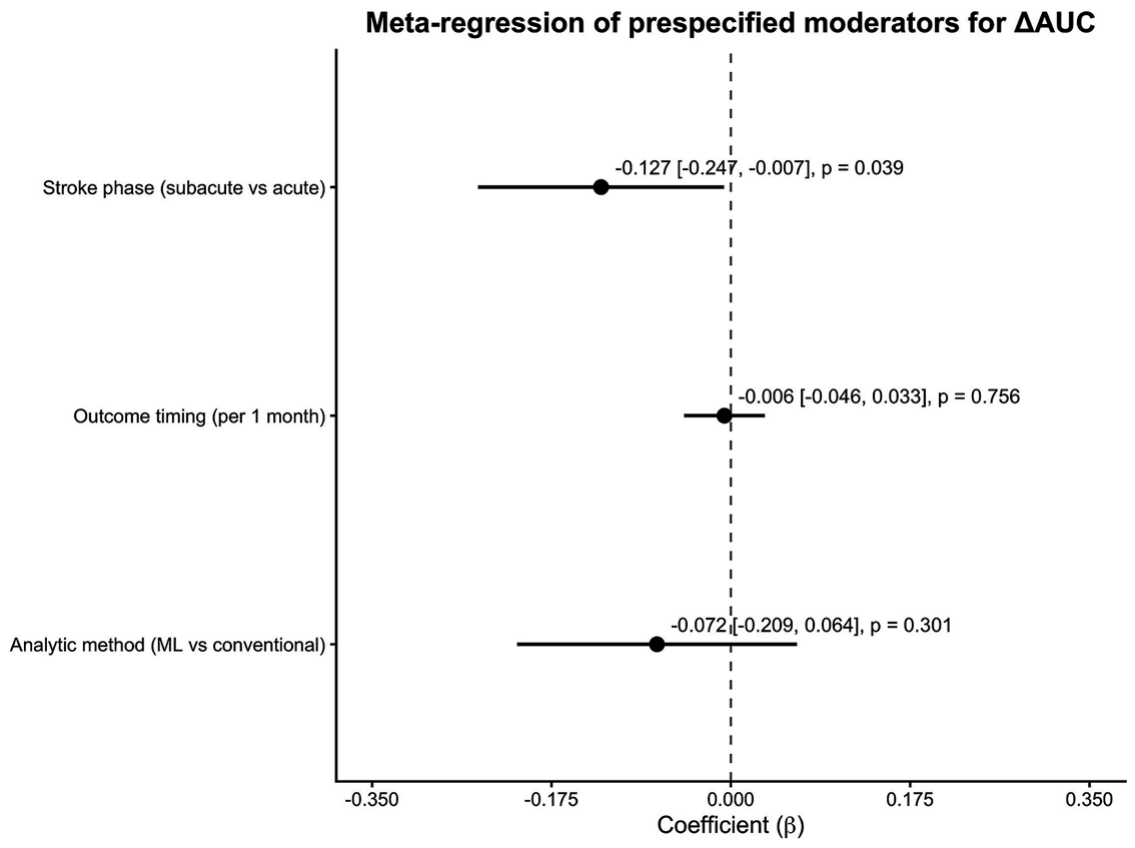
Meta-regression forest plot of prespecified moderator effects on incremental predictive value (ΔAUC). Points represent coefficient estimates for each moderator with corresponding 95% confidence intervals. The vertical dashed line at zero indicates no effect. Stroke phase was a statistically significant moderator (β = −0.127, 95% CI − 0.247 to −0.007, *p* = 0.039), whereas outcome timing (*β* = −0.006, 95% CI −0.046 to 0.033, *p* = 0.756) and analytic method (*β* = −0.072, 95% CI −0.209 to 0.064, *p* = 0.301) were not statistically significant. The model explained 44.76% of between-study heterogeneity (QM = 5.08, df = 3, *p* = 0.166).

### 3.6 Publication Bias

For the primary ΔAUC meta-analysis, funnel plot inspection and Egger’s test revealed no significant asymmetry (*p* = 0.278). Trim-and-fill analysis did not impute any missing studies (Figure 6). For the secondary ΔR^2^ analysis, funnel plot inspection suggested potential asymmetry. Trim-and-fill analysis imputed three hypothetical studies, adjusting the pooled ΔR^2^ from 0.175 to 0.064 (Supplementary Figure 3), suggesting possible publication bias. Given the small number of studies (*k* = 7), this finding should be interpreted cautiously.

**Figure 6:**
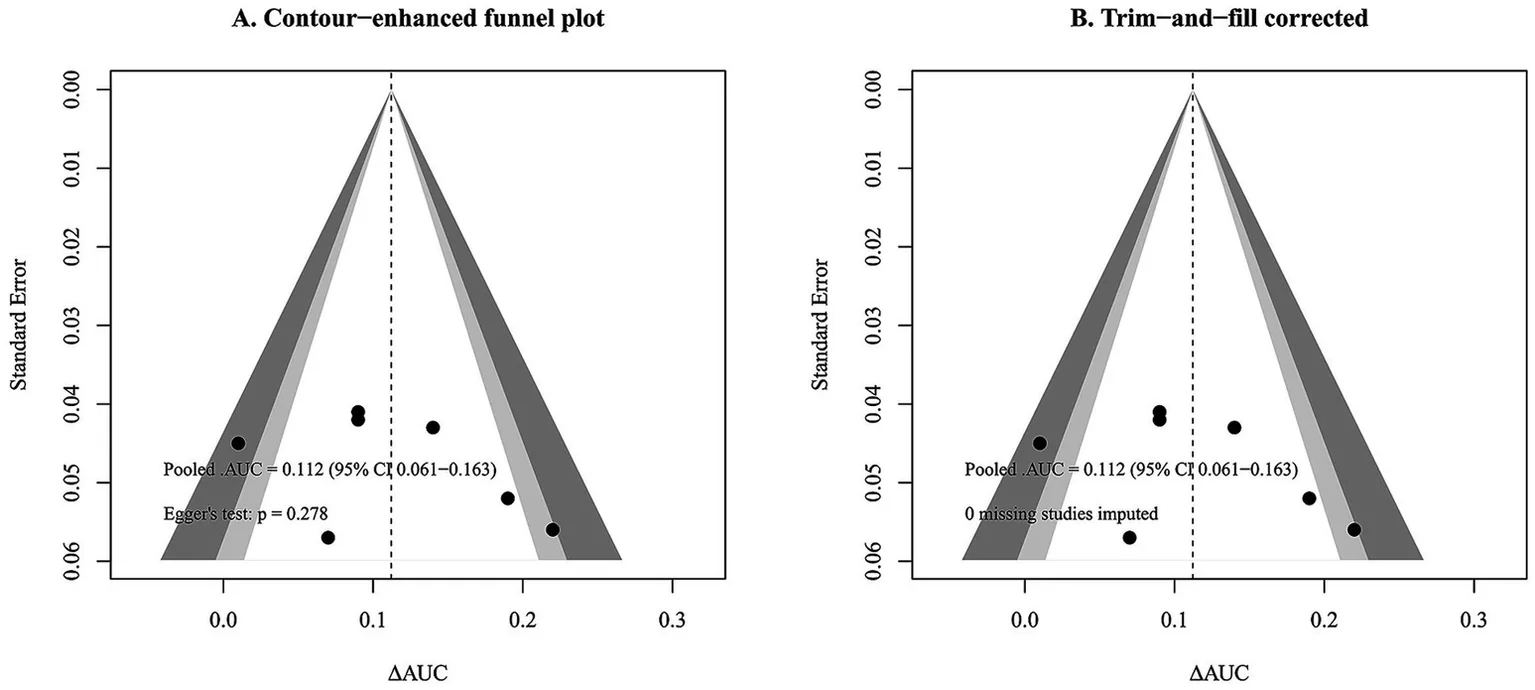
Funnel plots for assessment of publication bias in the primary ΔAUC meta-analysis. **(A)** Contour-enhanced funnel plot of the 7 included studies. Shaded regions correspond to contour levels of statistical significance (0.10 < *p* ≤ 1.00; 0.05 < *p* ≤ 0.10; 0.01 < *p* ≤ 0.05; 0.00 < *p* ≤ 0.01). Egger’s test revealed no significant asymmetry (*p* = 0.278), and the pooled random-effects estimate was ΔAUC = 0.112 (95% CI 0.061–0.163). **(B)** Trim-and-fill corrected funnel plot. No missing studies were imputed, confirming the robustness of the primary estimate. Black circles represent observed studies; no imputed studies were added. The vertical dashed line indicates the pooled random-effects estimate (ΔAUC = 0.112, 95% CI 0.061–0.163).

### 3.7 Sensitivity analysis

We performed a series of sensitivity analyses to assess the robustness of the primary ΔAUC estimate, with all results presented in Supplementary Table 3 (unless otherwise specified). Leave-one-out analysis showed that the pooled ΔAUC remained stable, ranging from 0.097 to 0.129 after sequential omission of each study (Supplementary Table 2; Supplementary Figure 4). The use of alternative between-study variance estimators produced similar pooled estimates (REML: 0.112, 95% CI 0.061 to 0.163; Paule-Mandel: 0.112, 95% CI 0.060 to 0.165). After Hartung-Knapp adjustment for small-sample confidence intervals, the pooled ΔAUC was 0.112 (95% CI 0.047 to 0.177), with a prediction interval of −0.026 to 0.250. The fixed-effects model produced a pooled estimate of 0.109 (95% CI 0.074 to 0.143). Excluding studies with high risk of bias in ≥2 QUIPS domains and restricting to studies reporting ΔAUC directly both produced a pooled estimate of 0.112 (95% CI 0.061 to 0.163). Heterogeneity remained unchanged (*I*^2^ = 52.4%). Overall, the point estimate and its confidence interval remained consistently positive across all sensitivity analyses; however, the prediction interval crossed zero (−0.026 to 0.250), suggesting that the true effect in future settings could be null or negative.

## 4 Discussion

### 4.1 Principal findings

This systematic review and meta-analysis quantified the incremental predictive value of adding biomarkers to clinical assessment for upper limb motor recovery after stroke. The primary finding was that multimodal assessment improved discrimination over clinical-only models, with a pooled ΔAUC of 0.112 (95% CI 0.061 to 0.163). Among biomarker subgroups, MEP status provided the largest pooled incremental gain (ΔAUC = 0.150), whereas ipsilesional CST FA was reported in a single study and other DTI-derived metrics did not reach statistical significance. Meta-regression identified stroke phase as a statistically significant moderator, with larger incremental value in the subacute phase. For the secondary outcome, the pooled ΔR^2^ was 0.175 (95% CI 0.082 to 0.269), with a prediction interval of −0.072 to 0.422. These findings extend previous narrative reviews by providing the first pooled estimate of incremental predictive value in this field. However, given the limited number of studies and the prediction intervals crossing zero for both ΔAUC and ΔR^2^, these results should be interpreted as statistically promising rather than definitive.

### 4.2 Biomarker-specific incremental value

Among the biomarkers assessed, MEP status had the largest pooled incremental gain. This finding is consistent with previous evidence that preserved MEP responses are associated with favorable motor outcomes (10, 12, 35). However, previous studies did not quantify the incremental predictive value of adding MEP to clinical models. Our pooled ΔAUC of 0.150 provides a quantitative estimate of this incremental value. Ipsilesional CST FA was reported in only one study, with a ΔAUC of 0.220, but pooled analysis was not possible; this finding requires confirmation in larger cohorts. Other DTI-derived metrics were heterogeneous and did not show a statistically significant pooled incremental gain. These findings suggest that, at present, MEP status represents the most promising biomarker, although its incremental value requires further validation in larger prospective cohorts before clinical application.

### 4.3 Sources of heterogeneity

Meta-regression identified stroke phase as a significant moderator, with larger incremental value of multimodal assessment in the subacute phase (7–90 days) than in the acute phase (<7 days). Outcome timing and analytic method were not significant moderators. Although the model explained a substantial proportion of between-study heterogeneity, the overall test of moderators was not statistically significant, indicating that stroke phase carries an independent effect while other prespecified moderators contribute little; this model should therefore be interpreted with caution (8, 35–37).

The larger incremental value in the subacute phase is biologically plausible. The subacute period is characterized by heightened neuroplasticity, when activity-dependent reorganization and corticospinal remodeling are most active (24, 27). During this window, biomarkers that capture residual corticospinal integrity—such as MEP status and CST FA—may add more prognostic information beyond clinical scales alone, because clinical scores at this stage are still evolving and less stable. In contrast, in the acute phase, clinical assessment may already reflect severe impairment, leaving less room for biomarkers to improve discrimination. However, only two of the seven studies contributing to the primary ΔAUC analysis were conducted in the subacute phase, and both had relatively small sample sizes. Therefore, this finding should be considered hypothesis-generating and requires confirmation in larger studies with balanced representation of acute and subacute phases.

The remaining heterogeneity may be attributable to differences in study populations, stroke type, clinical model definitions, outcome measures, follow-up timing, biomarker metrics, and modeling approaches (14, 18, 26). These methodological differences highlight the need for standardized definitions and reporting in future studies to facilitate more robust pooled analyses.

Direct evidence linking biomarker-based stratification to improved rehabilitation outcomes remains limited, and prospective validation in diverse cohorts is urgently needed (24, 27). In particular, future studies should test whether the larger incremental value observed in the subacute phase translates into improved patient selection for intensive rehabilitation or neuromodulation.

### 4.4 Comparison with previous reviews

Previous reviews have called for multimodal integration in stroke recovery prediction (2, 5, 18), and the SRRR consensus has recommended standardized biomarker measurement (24, 27). However, these efforts did not provide pooled estimates of incremental predictive value. The present study provides a pooled ΔAUC estimate for multimodal versus clinical-only models and reports biomarker-specific results.

For MEP status, previous studies have consistently reported that preserved MEP responses are associated with favorable motor outcomes (10, 12, 35), but these studies did not quantify the incremental value of adding MEP to clinical models. However, the predictive value of MEPs is not uniform across all studies, and methodological heterogeneity—including variations in the timing of MEP assessment and the target muscles selected—has been identified as a major source of inconsistent results (11, 38). In our analysis, the MEP subgroup also showed considerable heterogeneity, which may be partly attributable to methodological differences in the timing of MEP assessment and the target muscles selected, as both contributing studies were conducted in the acute phase. Our pooled ΔAUC of 0.150 provides the first quantitative estimate of the incremental value of MEP status, and it aligns with recent reviews indicating that MEP presence or absence remains the most established neurophysiological biomarker for upper limb recovery prediction (39, 40).

For DTI-derived metrics, previous studies have reported that CST integrity predicts motor recovery (7, 8, 35). However, the specific DTI metrics used across studies are not equivalent. In our analysis, only one study reported ipsilesional CST FA, with a ΔAUC of 0.220; because pooled analysis was not possible, this estimate should be interpreted as preliminary evidence, requiring external validation in larger cohorts (11, 37). This finding contrasts with previous reviews that have listed CST FA as one of the most established biomarkers (11, 18, 39); our results suggest that the current evidence base for CST FA is still limited. Other DTI-derived metrics (FAAI/rFA, LDH, lesion load, whole-brain volumes) were reported in three studies, with a pooled ΔAUC of 0.074 (95% CI − 0.006 to 0.155) that did not reach statistical significance. Previous studies have reported mixed results for these metrics (9–11). This finding is consistent with recent reviews indicating that not all DTI-derived metrics are equivalent to CST FA (39), and that pooled analysis should distinguish between them.

For task-based fMRI, previous reviews have highlighted its potential for predicting motor recovery (14, 15). However, in our analysis, only one study reported ΔAUC (0.190) together with a clinical-only comparator. Most fMRI studies did not report ΔAUC or a clinical-only comparator, and therefore could not be included in the pooled analysis (14, 15, 18). This finding highlights the need for standardized reporting of incremental predictive value in future fMRI studies.

Similarly, EEG, kinematic parameters, and resting-state fMRI could not be pooled because most studies did not report ΔAUC or a clinical-only comparator. This mirrors concerns raised in prior reviews regarding the heterogeneity of outcome metrics and the lack of standardized reporting in this field (14, 18).

### 4.5 Sensitivity analyses and robustness

The consistency of the primary estimate across sensitivity analyses is particularly important given the small number of studies. In meta-analyses with few studies, standard confidence intervals based on normal approximation may be too narrow, and the Hartung-Knapp adjustment provides more reliable inference (41). Simulation studies have demonstrated that when heterogeneity is present and the number of studies is small, the Hartung-Knapp correction outperforms the DerSimonian-Laird method, appropriately widening the confidence intervals to reflect more accurately the uncertainty around the pooled estimate (41, 42).

In our analysis, although the point estimate and its confidence interval remained consistently positive, and the leave-one-out range (0.097 to 0.129) indicated that the pooled estimate was not driven by any single study, the prediction interval crossed zero. This indicates that the true effect in future settings could be null or even negative, highlighting the uncertainty inherent in the current evidence base. Therefore, while the point estimate is statistically significant, the clinical implications must be interpreted with substantial caution, and the findings should be viewed as hypothesis-generating rather than definitive (43). Biomarker-guided stratification may still hold clinical potential, but future studies with larger sample sizes and standardized biomarker measurement are needed to narrow the prediction interval and confirm these findings.

### 4.6 Limitations

Several limitations should be acknowledged. First, only seven studies contributed to the primary ΔAUC analysis, and only two contributed to the MEP subgroup. Although meta-regression identified stroke phase as a significant moderator, the small number of studies limited the statistical power to detect other potential moderators. Moreover, only two of the seven studies contributing to the primary ΔAUC analysis were conducted in the subacute phase (9), so the significant effect of stroke phase should be interpreted with caution. Second, ipsilesional CST FA was reported in a single study, precluding pooled analysis. Third, other DTI-derived metrics were heterogeneous and did not reach statistical significance. Fourth, EEG, kinematic parameters, and resting-state fMRI could not be pooled because most studies did not report ΔAUC or a clinical-only comparator, rather than because of a gap in the search strategy. Fifth, most included studies enrolled moderate-to-severe ischemic stroke patients, limiting generalizability to mild strokes or hemorrhagic populations. Sixth, the small number of chronic-phase studies precluded quantitative synthesis for late recovery. Seventh, most studies were conducted in high-income countries, limiting generalizability to low- and middle-income settings (44). Eighth, most included studies did not report pre-stroke socioeconomic status, which has been shown to predict upper limb motor recovery (45) and may act as an unmeasured confounder of biomarker performance. However, because socioeconomic status was not consistently reported across studies, we could not evaluate its impact on the pooled estimates. Ninth, the definitions of “clinical-only” models varied across studies, which may have contributed to the observed heterogeneity (*I*^2^ = 52.4% for ΔAUC and 91.8% for ΔR^2^); residual heterogeneity remained despite sensitivity analyses. Finally, the pooled estimates should be interpreted with caution given the heterogeneity and the observational nature of the included studies (46).

### 4.7 Implications for future research

Future studies should report ΔAUC consistently and include a clinical-only baseline model. Standardized reporting of incremental predictive value metrics is needed to enable meta-analysis across biomarkers, particularly for those currently precluded from pooled analysis due to insufficient reporting (e.g., EEG, kinematic parameters, and resting-state fMRI) (14, 18). Longitudinal multimodal sampling at standardized time points may help characterize biomarker trajectories. Given that stroke phase significantly moderated the incremental value in our analysis, future studies should specifically evaluate whether biomarker-guided stratification is most effective in the subacute phase, and whether this translates into improved rehabilitation outcomes. External validation of MEP status and CST FA in diverse multicenter cohorts is needed before these biomarkers can be used for prognostic stratification.

From a clinical perspective, MEP status represents a promising candidate for prognostic stratification, but the current evidence base does not yet support its routine use in clinical practice. Prospective clinical trials are needed to evaluate whether MEP-guided stratification translates into improved rehabilitation outcomes.

### 4.8 Conclusion

In this systematic review and meta-analysis, adding selected biomarkers to clinical assessment was associated with improved discrimination for upper limb motor recovery after stroke (pooled ΔAUC = 0.112, 95% CI 0.061 to 0.163). However, given the small number of studies and the prediction interval crossing zero, this finding should be considered statistically promising but not yet robust enough to guide routine clinical practice. MEP status showed the largest incremental gain, whereas ipsilesional CST FA was reported in only one study and other DTI-derived metrics were not significant. Although several biomarkers (e.g., EEG, kinematic parameters) could not be pooled due to insufficient reporting, stroke phase significantly moderated the incremental value, with larger gains in the subacute phase. Future large-scale, multicenter studies are urgently needed to confirm these findings before clinical implementation.

## Data Availability

The original contributions presented in the study are included in the article/Supplementary material, further inquiries can be directed to the corresponding author.

## Supplementary material

SUPPLEMENTARY FIGURE 1 Summary of QUIPS risk of bias across the 23 included studies. Bars show the number of studies rated as low (green), moderate (yellow), and high (red) risk of bias in each of the six QUIPS domains. The x-axis indicates the number of studies. Three studies were rated as high risk of attrition bias. Abbreviation: QUIPS, Quality in Prognosis Studies.

SUPPLEMENTARY FIGURE 2 Study-level QUIPS risk of bias assessment for the 23 included studies. Each row represents one study, and each column represents one of the six QUIPS domains. Green = low risk, yellow = moderate risk, red = high risk. Three studies were rated as high risk of attrition bias, consistent with the domain summary in Supplementary Figure 1 and the detailed ratings in Supplementary Material 3. QUIPS, Quality in Prognosis Studies.

SUPPLEMENTARY FIGURE 3 Funnel plots for assessment of publication bias in the secondary ΔR^2^ meta-analysis. (A) Original funnel plot of the 7 included studies. The red solid line indicates the pooled random-effects estimate (ΔR^2^ = 0.175, 95% CI 0.082–0.269). (B) Trim-and-fill corrected funnel plot. Three hypothetical studies were imputed (open circles); the red solid line indicates the adjusted pooled estimate (ΔR^2^ = 0.064), and the dashed line indicates the original pooled estimate (ΔR^2^ = 0.175). Given the small number of studies (k = 7), this finding should be interpreted cautiously.

SUPPLEMENTARY FIGURE 4 Leave-one-out sensitivity analysis for the primary ΔAUC meta-analysis. Each row represents the pooled ΔAUC estimate after sequential omission of the indicated study. The vertical dashed line indicates no incremental predictive value (ΔAUC = 0). The overall pooled estimate including all 7 studies is ΔAUC = 0.112 (95% CI 0.061–0.163). Pooled estimates ranged from 0.097 to 0.129, indicating that the primary estimate was not driven by any single study.

SUPPLEMENTARY MATERIAL 1 Complete search strategies for PubMed, Embase, and Web of Science, including exact strings and number of records retrieved.

SUPPLEMENTARY MATERIAL 2 Detailed QUIPS domain ratings for all 23 included studies, including domain-level assessments, supporting evidence, review-specific considerations, and inter-rater reliability.

SUPPLEMENTARY MATERIAL 3 Extracted effect sizes for all included studies, conversion formulas for different statistical metrics (OR, R², t, F to AUC or Fisher’s z), and datasets used for meta-analyses.

SUPPLEMENTARY MATERIAL 4 Complete R code for all meta-analyses, meta-regression, publication bias assessment, and figure generation, ensuring full reproducibility.

SUPPLEMENTARY TABLE 1 Characteristics of the 23 included studies, including study design, patient demographics, stroke characteristics, prediction modalities, outcome measures, and model performance metrics.

SUPPLEMENTARY TABLE 2 Leave-one-out sensitivity analysis for the primary ΔAUC meta-analysis, showing pooled estimates after omitting each individual study.

SUPPLEMENTARY TABLE 3 Sensitivity analyses for the primary ΔAUC meta-analysis, including alternative between-study variance estimators, Hartung-Knapp adjustment, fixed-effects model, and risk-of-bias exclusion.
