# Supplementary material for: Incremental predictive value of multimodal over unimodal assessment for upper limb motor recovery after stroke: a systematic review and meta-analysis
Source: Front Neurol. 2026 Oct 28;17:1845257. doi: 10.3389/fneur.2026.1845257 (PMC13751340; doi:10.3389/fneur.2026.1845257)
Supplement: Supplementary file 1 [file Supplementary_file_4.DOCX]

**Supplementary Material 4. Complete R code for all meta-analyses**

\# ============================================================

\# sensitivity analyses, publication bias assessment,

\# and figure generation

\#

\# Manuscript: Incremental Predictive Value of Multimodal over

\#	Unimodal Assessment for Upper Limb Motor Recovery

\#	after Stroke: A Systematic Review and Meta-Analysis

\# PROSPERO: CRD420261288506

\# R version: 4.3.1

\# Package: metafor (and ggplot2 for Figure 5)

\# ============================================================

\# 0. Load packages

\# install.packages("metafor")

\# install.packages("ggplot2")

library(metafor)

library(ggplot2)

\# ============================================================

\# 1. Primary ΔAUC dataset (7 studies)

\# ============================================================

dat_auc <- data.frame(

study = c("Hoonhorst 2018", "Tao 2022", "Kumar 2022",

"Liu G 2017", "Park 2022", "Du 2018", "Liu G 2022"),

yi = c(0.09, 0.07, 0.22, 0.14, 0.01, 0.19, 0.09),

sei = c(0.042, 0.057, 0.056, 0.043, 0.045, 0.052, 0.041),

stroke_phase = c("acute", "subacute", "acute", "acute",

"subacute", "acute", "acute"), # Tao 2022 corrected

outcome_timing = c(6, 3, 6, 3, 3, 3, 3), # 7 values

analytic_method = c("conventional", "conventional", "conventional",

"conventional", "conventional", "conventional", "ML"),

biomarker = c("MEP", "Other DTI", "MEP", "Other DTI",

"Other DTI", "fMRI", "Other DTI")

)

dat_auc$vi <- dat_auc$sei^2

\# ============================================================

\# 2. Primary ΔAUC meta-analysis

\# ============================================================

res_DL	<- rma(yi, vi, data = dat_auc, method = "DL")

res_REML <- rma(yi, vi, data = dat_auc, method = "REML")

res_PM	<- rma(yi, vi, data = dat_auc, method = "PM")

res_HKSJ <- rma(yi, vi, data = dat_auc, method = "REML", test = "knha")

res_FE	<- rma(yi, vi, data = dat_auc, method = "FE")

summary(res_DL)

summary(res_REML)

summary(res_PM)

summary(res_HKSJ)

summary(res_FE)

pred_int <- predict(res_HKSJ)

print(pred_int)

\# ============================================================

\# 3. Leave-one-out sensitivity analysis

\# ============================================================

loo <- leave1out(res_DL)

print(loo)

\# ============================================================

\# 4. Subgroup analyses by biomarker type

\# ============================================================

\# MEP

res_MEP <- rma(yi, vi,

data = subset(dat_auc, biomarker == "MEP"),

method = "DL")

summary(res_MEP)

\# Ipsilesional CST FA (single study, S09)

res_CSTFA <- rma(yi, vi,

data = subset(dat_auc, study == "Kumar 2022"),

method = "DL")

summary(res_CSTFA)

\# Other DTI-derived metrics

res_OtherDTI <- rma(yi, vi,

data = subset(dat_auc, biomarker == "Other DTI"),

method = "DL")

summary(res_OtherDTI)

\# Task-based fMRI (single study, S13)

res_fMRI <- rma(yi, vi,

data = subset(dat_auc, biomarker == "fMRI"),

method = "DL")

summary(res_fMRI)

\# ============================================================

\# 5. Meta-regression

\# ============================================================

res_meta <- rma(yi, vi,

mods = ~ stroke_phase + outcome_timing + analytic_method,

data = dat_auc)

summary(res_meta)

\# Sensitivity meta-regression excluding outcome_timing

res_meta_sens <- rma(yi, vi,

mods = ~ stroke_phase + analytic_method,

data = dat_auc)

summary(res_meta_sens)

\# ============================================================

\# 6. Publication bias

\# ============================================================

\# Egger's test (primary ΔAUC)

regtest(res_DL)

\# Trim-and-fill (primary ΔAUC)

tf_auc <- trimfill(res_DL)

summary(tf_auc)

\# ============================================================

\# 7. Secondary ΔR² dataset (7 studies)

\# ============================================================

dat_r2 <- data.frame(

study = c("Jeong 2023", "Kim 2025", "Park 2022",

"Lim 2020", "Nemati 2022", "Hannanu 2017", "Kalinosky 2013"),

yi = c(0.08, 0.32, 0.01, 0.01, 0.24, 0.31, 0.37),

sei = c(0.024, 0.056, 0.026, 0.022, 0.046, 0.071, 0.076)

)

dat_r2$vi <- dat_r2$sei^2

res_r2_DL <- rma(yi, vi, data = dat_r2, method = "DL")

summary(res_r2_DL)

pred_int_r2 <- predict(res_r2_DL)

print(pred_int_r2)

tf_r2 <- trimfill(res_r2_DL)

summary(tf_r2)

\# ============================================================

\# 8. Sensitivity analysis summary table (Supplementary Table 3)

\# ============================================================

sens_table <- data.frame(

Analysis = c("Primary (DL)",

"REML",

"Paule-Mandel",

"REML + Hartung-Knapp",

"Fixed-effects",

"Excluding high risk of bias",

"Only direct AUC studies",

"Leave-one-out (range)"),

Pooled = c(sprintf("%.3f (%.3f-%.3f)", res_DL$b, res_DL$ci.lb, res_DL$ci.ub),

sprintf("%.3f (%.3f-%.3f)", res_REML$b, res_REML$ci.lb, res_REML$ci.ub),

sprintf("%.3f (%.3f-%.3f)", res_PM$b, res_PM$ci.lb, res_PM$ci.ub),

sprintf("%.3f (%.3f-%.3f)", res_HKSJ$b, res_HKSJ$ci.lb, res_HKSJ$ci.ub),

sprintf("%.3f (%.3f-%.3f)", res_FE$b, res_FE$ci.lb, res_FE$ci.ub),

sprintf("%.3f (%.3f-%.3f)", res_DL$b, res_DL$ci.lb, res_DL$ci.ub),

sprintf("%.3f (%.3f-%.3f)", res_DL$b, res_DL$ci.lb, res_DL$ci.ub),

sprintf("%.3f-%.3f", min(loo$estimate), max(loo$estimate))),

I2	= c(sprintf("%.1f%%", res_DL$I2),

sprintf("%.1f%%", res_REML$I2),

sprintf("%.1f%%", res_PM$I2),

sprintf("%.1f%%", res_HKSJ$I2),

sprintf("%.1f%%", res_FE$I2),

sprintf("%.1f%%", res_DL$I2),

sprintf("%.1f%%", res_DL$I2),

"—"),

tau2 = c(sprintf("%.4f", res_DL$tau2),

sprintf("%.4f", res_REML$tau2),

sprintf("%.4f", res_PM$tau2),

sprintf("%.4f", res_HKSJ$tau2),

sprintf("%.4f", res_FE$tau2),

sprintf("%.4f", res_DL$tau2),

sprintf("%.4f", res_DL$tau2),

"—"),

PI	= c("—", "—", "—",

sprintf("%.3f-%.3f", pred_int$pi.lb, pred_int$pi.ub),

"—", "—", "—", "—")

)

print(sens_table)

write.csv(sens_table, "Supplementary_Table_3_sensitivity.csv", row.names = FALSE)

\# ============================================================

\# 9. Figure 2. Forest plot of ΔAUC

\# ============================================================

pdf("Figure2_DeltaAUC_forest.pdf", width = 10, height = 5)

par(family = "serif", mar = c(5, 6, 4, 3))

forest(res_DL,

slab = dat_auc$study,

xlab = expression(Delta * "AUC"),

main = "Incremental predictive value (ΔAUC)",

cex.main = 1.1, font.main = 2,

refline = 0, digits = 3,

mlab = "Pooled ΔAUC")

dev.off()

\# ============================================================

\# 10. Figure 3. Forest plot of ΔR²

\# ============================================================

pdf("Figure3_DeltaR2_forest.pdf", width = 10, height = 5)

par(family = "serif", mar = c(5, 6, 4, 3))

forest(res_r2_DL,

slab = dat_r2$study,

xlab = expression(Delta * R^2),

main = "Incremental predictive value (ΔR²)",

cex.main = 1.1, font.main = 2,

refline = 0, digits = 3,

mlab = "Pooled ΔR²")

dev.off()

\# ============================================================

\# 11. Figure 4. Biomarker-specific forest plots

\# ============================================================

pdf("Figure4_biomarker_forest.pdf", width = 12, height = 10)

par(mfrow = c(2, 2), family = "serif",

mar = c(5, 6, 4, 3), oma = c(0, 0, 2, 0))

forest(res_MEP,

slab = dat_auc$study[dat_auc$biomarker == "MEP"],

xlab = expression(Delta * "AUC"),

main = "A. MEP status",

cex.main = 1.1, font.main = 2,

refline = 0, digits = 3,

mlab = "Pooled ΔAUC")

forest(res_CSTFA,

slab = "Kumar 2022 (CST FA)",

xlab = expression(Delta * "AUC"),

main = "B. Ipsilesional CST FA",

cex.main = 1.1, font.main = 2,

refline = 0, digits = 3,

mlab = "ΔAUC (single study, S09)")

forest(res_OtherDTI,

slab = dat_auc$study[dat_auc$biomarker == "Other DTI"],

xlab = expression(Delta * "AUC"),

main = "C. Other DTI-derived metrics",

cex.main = 1.1, font.main = 2,

refline = 0, digits = 3,

mlab = "Pooled ΔAUC")

forest(res_fMRI,

slab = "Du 2018",

xlab = expression(Delta * "AUC"),

main = "D. Task-based fMRI",

cex.main = 1.1, font.main = 2,

refline = 0, digits = 3,

mlab = "ΔAUC (single study, S13)")

dev.off()

\# ============================================================

\# 12. Figure 5. Meta-regression forest plot (ggplot2)

\# ============================================================

df <- data.frame(

moderator = factor(

c("Stroke phase (subacute vs acute)",

"Outcome timing (per 1 month)",

"Analytic method (ML vs conventional)"),

levels = c("Analytic method (ML vs conventional)",

"Outcome timing (per 1 month)",

"Stroke phase (subacute vs acute)")

),

beta = res_meta$b[2:4],

lower = res_meta$ci.lb[2:4],

upper = res_meta$ci.ub[2:4],

p	= res_meta$pval[2:4]

)

df$label <- sprintf("%.3f [%.3f, %.3f], p = %.3f",

df$beta, df$lower, df$upper, df$p)

p <- ggplot(df, aes(x = beta, y = moderator)) +

geom_vline(xintercept = 0, linetype = "dashed", color = "gray50") +

geom_segment(aes(x = lower, xend = upper,

y = moderator, yend = moderator),

linewidth = 0.8) +

geom_point(size = 3) +

geom_text(aes(label = label),

hjust = -0.05, vjust = -0.9, size = 3.5) +

scale_x_continuous(limits = c(-0.35, 0.35),

breaks = seq(-0.35, 0.35, 0.175)) +

labs(x = expression("Coefficient ("*beta*")"),

y = NULL,

title = "Meta-regression of prespecified moderators for ΔAUC") +

theme_classic(base_size = 12) +

theme(plot.title = element_text(hjust = 0.5, face = "bold"))

print(p)

ggsave("Figure5_meta_regression.pdf", p, width = 8, height = 6)

ggsave("Figure5_meta_regression.png", p, width = 8, height = 6, dpi = 600)

\# ============================================================

\# 13. Figure 6. Funnel plot for primary ΔAUC

\# ============================================================

pdf("Figure6_Publication_bias_assessment.pdf", width = 10, height = 6)

par(mfrow = c(1, 2), family = "serif",

mar = c(6, 6, 3, 2), oma = c(0, 0, 2, 0))

funnel(res_DL,

level = c(90, 95, 99),

shade = c("white", "gray75", "gray55", "gray35"),

refline = res_DL$b,

xlab = expression(Delta * "AUC"),

ylab = "Standard Error",

main = "A. Contour-enhanced funnel plot")

funnel(tf_auc,

level = c(90, 95, 99),

shade = c("white", "gray75", "gray55", "gray35"),

refline = tf_auc$b,

xlab = expression(Delta * "AUC"),

ylab = "Standard Error",

main = "B. Trim-and-fill corrected")

dev.off()

\# ============================================================

\# 14. Supplementary Figure 3. Funnel plot for ΔR²

\# ============================================================

pdf("Supplementary_Figure3_DeltaR2_funnel.pdf", width = 12, height = 6.5)

par(mfrow = c(1, 2), family = "serif",

mar = c(6, 6, 3, 2), oma = c(0, 0, 2, 0))

funnel(res_r2_DL,

level = c(90, 95, 99),

shade = c("white", "gray75", "gray55", "gray35"),

refline = res_r2_DL$b,

xlab = expression(Delta * R^2),

ylab = "Standard Error",

main = "A. Original funnel plot")

funnel(tf_r2,

level = c(90, 95, 99),

shade = c("white", "gray75", "gray55", "gray35"),

refline = tf_r2$b,

xlab = expression(Delta * R^2),

ylab = "Standard Error",

main = "B. Trim-and-fill corrected")

dev.off()

\# ============================================================

\# 15. Supplementary Figure 4. Leave-one-out forest plot

\# ============================================================

pdf("Supplementary_Figure4_leave_one_out.pdf", width = 10, height = 5)

par(family = "serif", mar = c(5, 7, 3, 2))

forest(loo$estimate,

sei = loo$se,

slab = paste0("Omit ", dat_auc$study),

xlab = expression(Delta * "AUC"),

header = c("Study omitted", "Pooled ΔAUC [95% CI]"),

cex = 1.0, cex.lab = 1.1, cex.axis = 1.0,

refline = 0, digits = 3,

col = "black", psize = 1)

mtext(sprintf("Overall pooled ΔAUC = %.3f (95%% CI %.3f-%.3f)",

res_DL$b, res_DL$ci.lb, res_DL$ci.ub),

side = 1, line = 3.5, adj = 0, cex = 0.85)

dev.off()

\# ============================================================

\# 16. Output files generated

\# ============================================================

cat("\nGenerated files:\n")

cat(" Figure2_DeltaAUC_forest.pdf\n")

cat(" Figure3_DeltaR2_forest.pdf\n")

cat(" Figure4_biomarker_forest.pdf\n")

cat(" Figure5_meta_regression.pdf / .png\n")

cat(" Figure6_Publication_bias_assessment.pdf\n")

cat(" Supplementary_Figure3_DeltaR2_funnel.pdf\n")

cat(" Supplementary_Figure4_leave_one_out.pdf\n")

cat(" Supplementary_Table_3_sensitivity.csv\n")
